# Three Approaches to Improve a Practical Guide on Evidence-Informed Deliberative Processes for Health Benefit Package Design: Comment on "Evidence-Informed Deliberative Processes for Health Benefit Package Design – Part II: A Practical Guide"

**DOI:** 10.34172/ijhpm.2022.7502

**Published:** 2022-08-31

**Authors:** Javier Guzman

**Affiliations:** Center for Global Development, Washington, DC, USA.

**Keywords:** Health Technology Assessment, Evidence-Informed Deliberative Processes, Legitimacy, Health Benefit Package

## Abstract

As countries around the world seek to deliver universal health coverage, they must prioritize which services to pay for with public funds, to whom, and at what cost. Countries are increasingly using health technology assessment (HTA) to identify which interventions provide the best value for money and merit inclusion in their health benefit packages (HBPs)—the explicit lists of health services provided using public funds. Oortwijn et al understand the importance of providing practical guidance on the foundation of HBP design, and their article, "Evidence-Informed Deliberative Processes for Health Benefit Package Design – Part II: A Practical Guide," provides recommendations for HTA bodies to improve the legitimacy of their decision-making by incorporating four elements in their HBP procedures: stakeholder involvement, evidence-informed evaluation, transparency, and appeal. This article proposes three approaches to enhance the value of the guide: moving from structure to compliance and performance, prioritizing key issues of legitimacy within HBP processes, and acknowledging potential the costs and risks associated with the use of this framework.

## Introduction

 As countries around the world seek to deliver universal health coverage, they must prioritize which services to pay with public funds, to whom, and at what cost. Countries are increasingly using health technology assessment (HTA) to identify which interventions provide the best value for money and merit inclusion in their health benefit packages (HBPs) — the explicit lists of health services and products provided using public funds.^1^ HBPs aim to provide the greatest impact on health outcomes, health equity and financial protection, given resources available and a country-specific burden of disease. HTA offers a systematic approach to measure the medical, economic, social, and ethical issues related to the use of a health technology and provides policy-makers with evidence-based information to support health policies that are safe, effective, and cost-effective.^2^

 Incorporating HTA into fair, legitimate processes for HBP design goes beyond developing know-how, establishing methods, and gathering data. It also requires creating sound processes and inserting them within adequate institutional arrangements and a strong legal framework. Such a framework would include clear legal provisions on when and how HTA will be used to inform healthcare decisions, how HTA will be conducted and by whom as well as legally binding rules on transparency, accountability, stakeholder participation and prevention and management of conflict of interests.^2^ Oortwijn et al understand the importance of providing practical guidance on the foundation of HBP design, and their article, “Evidence-Informed Deliberative Processes for Health Benefit Package Design – Part II: A Practical Guide,” provides recommendations for HTA bodies to improve the legitimacy of their decision-making by incorporating four elements in their HBP procedures: stakeholder involvement, evidence-informed evaluation, transparency, and appeal.^3^

 This article proposes three approaches to further enhance the value of the guide: moving from structure to performance, prioritizing key issues of legitimacy within HBP processes further, and acknowledging the potential costs and risks associated with the use of this framework.

## Approach 1. Moving From Structure to Compliance and Performance

 Oortwijn et al propose six steps to implement evidence-informed deliberative processes: installing an advisory committee, defining decision criteria, selecting health technologies for HTA, scoping, assessment and appraisal for every health technology, communications and appeal and monitoring and evaluation (M&E). Oortwijn et al make several recommendations on priority setting structures and processes within each step. For example, for the first step, installing an advisory committee, they provide detailed guidance on what the committee should do, who should be part of the committee, how members should be selected, and how the committee should reach its decisions. This guidance is useful for nascent and developing HTA bodies, but it should go one step further. The guide should ideally provide direction on how HTA bodies can measure and report on compliance and performance at each step of the HBP process. In other words, the guide should identify key indicators HTA bodies should measure to guarantee that the legal structures and processes established on paper get translated into actions, decisions, and realities. For example, HTA bodies would benefit from measuring key attributes such as transparency, impartiality, inclusivity, consistency, acceptance and even legitimacy of the HBP process.

 Oortwijn et al do suggest measuring as part of the M&E step in their guide, but only include two structural indicators that do not help HTA bodies monitor compliance and performance thoroughly: existence of a mechanism for M&E, and stakeholder involvement as part of the M&E process. Measuring whether adequate inputs and processes are in place is a necessary first step, but measuring whether those inputs are actually working and providing value is critical to delivering on the promises of a fairer, more effective, more legitimate and accepted HBP process that leads to better implementation and, ultimately, better health. Oortwijn has already worked to address this limitation and has recently published a Good Practices Report of a Joint HTAi/ISPOR Task Force with a M&E framework that includes process and outcome indicators.^4^

 The guide and the framework recently published could benefit from incorporating lessons learned from similar exercises, including the World Health Organization (WHO) Global Benchmarking Tool for evaluation of national regulatory systems.^5^ The Global Benchmarking Tool is relevant in providing a set of key tracer indicators that can help benchmark the HBP process, identifying strengths and areas for improvement, and facilitating the formulation of institutional development plans to build upon strengths and address identified gaps as well as monitoring progress.

## Approach 2. Prioritizing Key Issues of Legitimacy Within HBP Processes

 Oortwijn et al describe six steps in implementing evidence-informed deliberative processes (see steps listed in Approach 1 above), and within each step, they identify key issues of legitimacy, listed in the supplementary file attached to their manuscript. They end up with around 30 key issues, ranging from relatively minor matters such as duration of appraisal meetings to fundamental issues such as stakeholder involvement in the identification of health technologies for HTA. This approach, although comprehensive, does not provide clarity on how the listed issues relate to key aspects of legitimacy or what essential issues nascent and developing HTA bodies should prioritize to increase legitimacy overall. Additionally, the guide does not provide clear guidance or describe the advantages and disadvantages of how well-developed HTA bodies have made choices regarding these key issues listed. For example, nascent HTA bodies would benefit from additional clarity on the pros and cons of issuing binding decisions in relation to HBP legitimacy and uptake, as Germany and the United Kingdom do, compared to preparing advisory recommendations as done by other mature HTA bodies in Australia, Brazil, Canada, France, Scotland, and Thailand. Overall, including fewer issues in the guide but going deeper on priority issues might provide greater insights and be more useful for HTA practitioners than including more issues in a superficial, descriptive manner. Pragmatism and strategic thinking are needed to identify what is most important to increase the legitimacy of the HBP process.

## Approach 3. Acknowledging Costs and Risks Associated With Evidence-Informed Deliberative Processes

 Oortwijn et al acknowledge several limitations related to their analysis of how eight mature HTA bodies organize key aspects of legitimacy in their decision-making processes. However, their guide does not mention the costs and risks associated with incorporating and/or increasing stakeholder involvement, evidence-informed evaluation, transparency, and appeal in HBP processes, or in other words, establishing good governance principles.

 Working toward a transparent, participatory, con­sistent, and evidence informed HBP design process is costly as it requires expertise, institutional capacity, money, and time.^6^ These elements are in short supply, especially in lower-income countries where analytic and administra­tive capacity is limited, human resources are scarce, and govern­ments are skeptical of restricting their discretionary power. Increasing transparency and participation might also involve several tradeoffs, including reduced efficiency, flexibility, speed, creativity, empowerment, and innovation, and might even be counterproductive.^7^ For example, engaging patient groups with financial relationships with industry might lead to conflict of interests, potential threats to the integrity and independence of those groups, and undue support for industry positions. A systematic review of industry funding of patient and health consumer organizations found that industry funding of patient groups in high income countries is common, ranging from 20% to 83%, and that policies governing corporate sponsorship and transparency about corporate funding in patient groups are inadequate.^8^ The situation in low- and middle-income countries is likely to be worse as legal frameworks and enforcement practices are weaker or even lacking. Incorporating country lessons of positive but also negative experiences incorporating evidence-informed deliberative processes for HBP design will provide a balanced approach that will help policy-makers make informed decisions on the best way to improve HBP legitimacy and acceptance.

## Conclusion

 Oortwijn et al provide a valuable guide for improving the legitimacy of HBP design by incorporating four elements in HBP procedures: stakeholder involvement, evidence-informed evaluation, transparency, and appeal. Their guide can be further enhanced by moving from structure to compliance and performance, prioritizing key issues of legitimacy within HBP processes further, and acknowledging, in an explicit, clear, and realistic manner, the potential costs and risks associated with the use of this framework. Incorporating these elements in future iterations of the guide will help HTA bodies deliver on the promise of a fairer, more effective, more legitimate, and accepted HBP process that leads to better implementation and ultimately better health.

## Ethical issues

 Not applicable.

## Competing interests

 I was employed by the Colombian HTA Agency between 2013 and 2015. I also participated in a paid training session sponsored by a pharmaceutical industry in 2020.

## Author’s contribution

 JG is the single author of the paper.

## References

[1] Glassman A, Giedion U, Sakuma Y, Smith PC (2016). Defining a health benefits package: what are the necessary processes?. Health Syst Reform.

[2] Bertram M, Dhaene G, Tan-Torres Edejer T. Institutionalizing Health Technology Assessment Mechanisms: A How to Guide. Geneva: World Health Organization; 2021.

[3] Oortwijn W, Jansen M, Baltussen R (2022). Evidence-informed deliberative processes for health benefit package design-part II: a practical guide. Int J Health Policy Manag.

[4] Oortwijn W, Husereau D, Abelson J (2022). Designing and Implementing Deliberative Processes for Health Technology Assessment: A Good Practices Report of a Joint HTAi/ISPOR Task Force. Int J Technol Assess Health Care.

[5] Global benchmarking tool. World Health Organization website. https://www.who.int/tools/global-benchmarking-tools. Accessed August 4, 2022.

[6] Giedion U, Guzmán J. Defining the rules of the game: good governance principles for the design and revision of the health benefits package. In: Glassman A, Giedion U, Smith P, eds. What’s In, What’s Out: Designing Benefits for Universal Health Coverage. Washington, DC: Center for Global Development; 2017. p. 30-60.

[7] Greer SL, Wismar M, Figueras J. Strengthening Health System Governance: Better Policies, Stronger Performance. New York: Open University Press; 2016.

[8] Fabbri A, Parker L, Colombo C (2020). Industry funding of patient and health consumer organisations: systematic review with meta-analysis. BMJ.

